# Depression and its associated factors among prisoners in East Gojjam Zone prisons, Northwest Ethiopia: a multi-centered cross-sectional study

**DOI:** 10.1186/s40001-022-00766-0

**Published:** 2022-07-30

**Authors:** Tesfahun Tiruneh, Haile Amha, Mezinew Sintayehu Bitew, Yilkal Tafere

**Affiliations:** 1 Debre Markos Comprehensive Specialized Hospital, Debre Markos, Ethiopia; 2 Department of Nursing, Debre Markos University College of Health Science, P.O. Box: 269, Debre Markos, Ethiopia; 3 Department of Public Health, Debre Markos University College of Health Science, Debre Markos, Ethiopia

**Keywords:** Associated factors, Depression, Ethiopia, Prison

## Abstract

**Background:**

Little is known about the prevalence and risk factors for depression in this vulnerable population around the world, including Ethiopia. Furthermore, information on the health of inmates is limited. The study sought to assess the prevalence and associated factors for depression among prisoners in the East Gojjam Zone of Northwest Ethiopia.

**Methods:**

Institution-based cross-sectional study was conducted in East Gojjam Zone prisons. Data were gathered from 462 eligible prisoners who were chosen using a computer-generated simple random sampling technique. The patient health questionnaire nine was used to assess an individual's depression level. The information was entered into Epi-Data Version 4.2 and exported to STATA Version 14.1 for further analysis. Variables with a *P* < 0.05 in the multivariable binary logistics regression were considered significant.

**Results:**

In this study the prevalence of depression among prisoners was 50.43% (95% CI 46–55%). Having work inside prison (AOR 0.6, CI 0.37–0.96), have no history of mental illness (AOR 0.37, 95% CI 0.16–0.85), had monthly income greater than 1500 birr (AOR 0.16, CI 0.05–0.5), Not thinking about the life after prison (AOR 0.4, 95% CI 0.27–0.64), and Prisoners who are sentenced for more than 5 years (AOR 2.2, CI 1.2–4), were significantly associated with depression.

**Conclusions:**

According to this study, half of the prisoners in East Gojjam Zone prisons had depressive symptoms. Prisons should place a greater emphasis on the mental health of prisoners who have been sentenced for a long time, those who have a history of mental illness, and those who have no work in the prison.

**Supplementary Information:**

The online version contains supplementary material available at 10.1186/s40001-022-00766-0.

## Introduction

Depression is a psychiatric disease characterized by a gloomy mood, a lack of interest or pleasure in activities, and a loss of energy that lasts 2 weeks or longer. Symptoms may include changes in food, weight, sleep, and motoric activity, as well as feelings of triviality or guilt, difficulty thinking, concentrating, or making decisions, and persistent thoughts of death or suicide plots or attempts [1]. Depression entails more than simply feeling down or going through a difficult time. It is a major mental health problem that necessitates knowledge and treatment. Depression, if left untreated, may be disastrous for both the person suffering from it and their families. Mental diseases are prevalent in almost every country on the planet. In addition, it is estimated that 450 million people around the world suffer from mental illnesses [2, 3].

Several studies conducted in various countries consistently reported a gradual increase in depression morbidity across the general population. Depression affected over 350 million people worldwide. The severity of crime in prison far outweighs that of the general population. Prisoners all over the world are at a high risk of morbidity and victimization. Prisoners become depressed as a result of their living conditions, overcrowding, restrictions and lack of freedom, and other factors. Depression causes loss of freedom, insecurity about future prospects, insufficient health services, particularly mental health services, a lack of meaningful activities, a lack of social support, and interdependence [4–7].

When prisoners are afflicted with depression symptoms on a regular or chronic basis, it can lead to suicide. According to one study, one million people die each year as a result of suicide. This equates to 3000 deaths per day. Around one in every nine prisoners worldwide suffers from common mental health disorders, with depression symptoms being the most common. When compared to the general population, prisoners are 5 to 10 times more likely to suffer from depression [5, 8–12].

Various studies in various countries found that depressive symptoms are more prevalent in prisoners than in the general population. In Malaysia, 55.4% of prisoners were reported to be depressed [13]. In France, the prevalence of depression on male prisoners was 17.9% [14]. Prisoners in Iran (29%) [15] were depressed, as were those in Nigeria (42.2%) [16], Ethiopia Jimma (41.9%) [17], and Hawassa (56.4%) [8].

Despite the fact that more than two-third of incarcerated men and women live in low- and middle-income countries, the vast majority of evidence on mental disorders among prisoners is based on studies from high-income countries, providing implications that are hardly applicable or generalizable to low- and middle-income settings. The prevalence of psychiatric disorders in low-income countries’ penal justice systems may differ from that of high-income countries due to resource scarcity, cultural and legal factors [18].

In Sub-Saharan Africa, research on inmates’ mental health is limited, and largely focused on infectious disorders. Prisoners suffer from depression, which is a common public health issue. Inmates are frequently excluded from national health surveys, despite the fact that inmate populations are expanding, and information about prisoners’ mental health status in this subject area is scarce. Though there are some studies in the country, because prison administration varies from time to time and place to place, and this study specifically assessed depression, unlike other studies that focus on mental illness in general and identify factors related to depression.

## Methods

### Study design and period

From March 1 to March 30, 2019, an institution-based cross-sectional investigation was undertaken.

### Study area

The research was carried out in jails in the East Gojjam Zone. The Amhara Region of Ethiopia has 11 zones, one of which is the East Gojjam Zone. With an estimated population of 3,800,000 people, the zone encompasses a total area of 1292.98 km^2^. During the study period, three prisons in the East Gojjam Zone housed a total of 2648 inmates. Debre–Markos town is 298 km from Addis Ababa and 265 km from Bahir Dar; Bichena town is 265 km from Addis Ababa and 185 km from Bahir Dar; and Motta town is 370 km from Addis Ababa and 120 km from Bahir Dar.

### Study population

All prisoners found in East Gojjam Prisons available during the time of data collection period.

### Eligibility criteria

Prisoners who stayed in prison at least for 2 weeks and do not have severe illnesses.

### Procedure for determining sample size

The maximum required sample size was determined by balancing two objectives. The sample size for the first objective was calculated using a single population proportion formula while taking into account the following statistical assumptions: 95% confidence interval, power, 80%, and 5% marginal error. Depression prevalence (*P* = 44%) was derived from a previous study [19]

$$[n = [(Za/2)^2 * p(1-p)]/d^2.$$

The calculated value of the initial sample size was 37 using the above formula. The sample size for the second objective was calculated using the Epi info statistical package version 7 software by considering the prior stud’s significant factors. Power 80% and CI 95% were utilized as assumptions. As a result of the assumption, the highest needed sample size estimated with Epi information was 422. The total sample size was 464 after accounting for a 10% nonresponse rate (Table 1).

**Table 1:** Sample size determination using Epi info statcalc for prevalence and associated factors of depression among prisoners in East Gojjam Zone prison, Amhara, and Northwest Ethiopia 2019

Variables	Depression		Sample size	10% none response rate
	Exposed (%)	Unexposed (%)		
Impossibilities to run life after prison [19]	58	36.6	188	207
Had plan to commit suicide [19]	75	37	62	68
Social support [19]	37	51	422	464

**Fig. 1:**
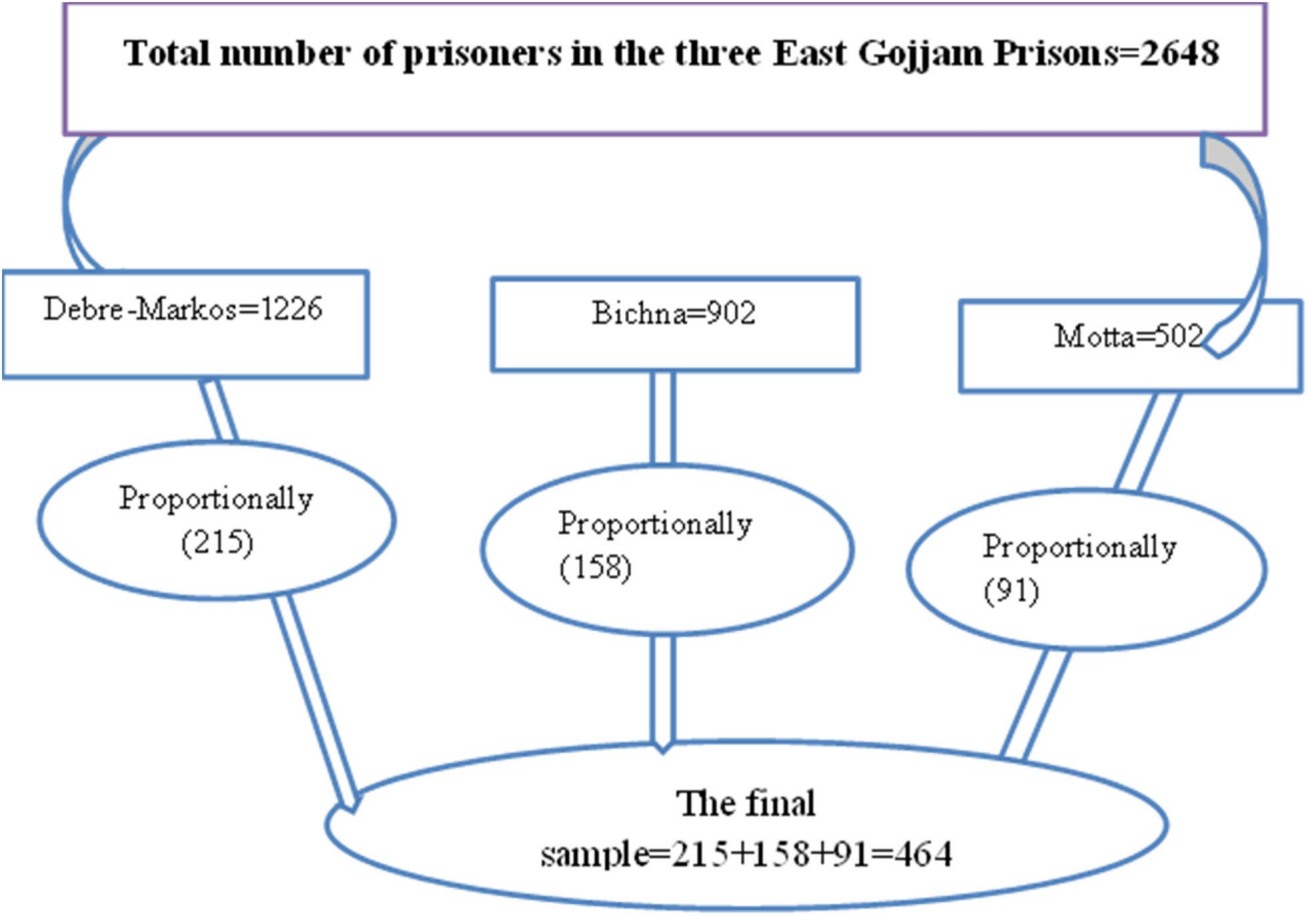
Sample size proportional allocation for prevalence and associated factors of depression among prisoners in East Gojjam Zone prison, Amhara Region, Ethiopia 2019

A proportional fraction of the total sample size was given to each prison. The formula *n*_*i *_ = *N*_*i *_*n/N* was applied for the three jails. The study participants in each prison were then picked using a simple random sampling technique utilizing a computer-generated random sample technique (Fig. 1).

### Data collection tools and procedure

Data were collected by five trained psychiatry nurses using structured questionnaire through face-to-face interview method (Additional file 1). Questionnaires had four parts, which are socio-demographic characteristics, environmental conditions of prisoners in prison, Patient health questionnaire nine (PHQ9), which contained nine questions each measuring a problem that the prisoners bothered in the last 15 days was used to measure depression with scale measurement ranging from 0 (not at all) to 3 (nearly every day) and clinical factors assessment tool.

PHQ-9 is a validated and widely used tool used to assess depression in Ethiopia in clinical and community settings [20–22]. The PHQ-9 has good reliability and validity [sensitivity (86%) and specificity (67%)] for diagnosing MDD among Ethiopian adults. The internal consistency reliability was also found to be excellent 0.92 and Cronbach’s alpha of 0.81. A threshold of ten on the PHQ-9 was the most appropriate cut off and offered the optimal discriminatory power in detecting depression [23]. This tool also used by different studies previously on inmates in Ethiopian and its reliability is confirmed [24–26].

Receiver operating characteristics curve (ROC) analysis was done using STATA version 14.1software to cheek the recommended cut of value with maximum sensitivity and specificity. An individual was considered as in a state of depression if he or she scores 10 and above. Within our data the ROC area was 0.99935 at cutoff 10. The tool validation test was also done in Ethiopia [21] and cut off value ≥ 10 to screen depressive symptoms is recommended by different literatures [27–29]. OSLO-3 which contains three items was used to assess the social support status [21]. Using the OSLO-3 scale, an individual is deemed to have weak social support if he or she scores 3–8, moderate social support if he or she scores 9–11, and good social support if he or she scores 12–14 [30].

### Data processing and analysis

Epi-Data version 4.2 was used to enter the data, which was cheeked for completeness and consistency. The data was then exported to the STATA statistical program version 14.1 for additional analysis. Tables, graphs, and texts were used to calculate and describe descriptive statistics, such as frequency tables. The binary logistic regression models were used to discover factors linked to depression among jail inmates. The multivariable logistic regression model was fitted with variables that had a *P* value of less than 0.25 in the bivariable analysis. The Hosmer and Lemshow goodness of fit test was used to assess model fitness, yielding a *P* value of 1.15. Finally, in multivariable analysis, variables with a *P* value less than 0.05 at the 95% confidence range were judged to be factors substantially associated with depression.

### Data quality control

To assure data quality, pretest was done among 5% (22 participants) of a total respondent who are randomly selected from Finot–Selam town prisoners check the understandability and reliability of the questionnaires. The questionnaire is written in English and then translated into Amharic, and back to English language to check consistency of the questionnaire. Two days training for five data collectors and three supervises was given on the study instrument, data collection procedure and the ethical principles of confidentiality. The collected data was reviewed and checked for completeness and relevance by the supervisors and principal investigator each day.

### Ethical consideration

The ethical clearance was obtained from the ethical review committee of the University’s Health Science College. Permission letters were also obtained from each of the three prison administration offices. Each study participant provided informed verbal consent prior to data collection. Furthermore, the information gathered from the respondent prisoners was used solely for research purposes and was kept strictly confidential. Furthermore, the names of study participants were not included to maintain confidentiality. Prisoners who became severely depressed during the interview were referred to health services and contacted by guidance counseling personnel at the prison.

## Results

A total of 464 prisoners were sampled to be included in the study, and 462 (99.5%) agreed to participate. The respondents’ median age was 30 years, with an interquartile range (IQR = 24–40) years. The majority of the respondents, 411 (88.96%) were men. The majority of those who responded were Orthodox Christians (95.88%). From a total of 462 respondents, 123 (26.62%) could not read or write and 22 (4.76%) had a diploma or higher educational level. Three hundred nine (66.88%) of the 462 respondents were from rural areas. The majority of the 452 respondents (97.84%) were Amhara by ethnicity. More than half 274 (59.31%) of study participants were married. In addition, 276 (59.74%) had children (Table 2).

**Table 2:** Socio-demographic characteristics of study participants of prisoners in East Gojjam Zone prisons, Northwest Ethiopia, 2019 (*N* = 462)

Socio demographic study variables of respondents	Frequency (*N*)	Percentage
Sex		
Male	411	88.6
Female	51	11.4
Age		
18–34 years	290	62.77
35–49 years	118	25.54
≥ 50 years	54	11.69
Religion		
Orthodox	443	95.89
Others	19	4.11
Residency		
Urban	152	33.19
Rural	310	66.81
Ethnicity		
Amhara	452	97.86
Others	10	2.14
Marital status		
Married	274	59.27
Unmarried	117	25.43
Divorced	62	13.36
Widowed	9	1.94%
Having children		
Yes	276	59.74
No	186	49.26
Educational status		
Unable to read and write	94	20.35
1–8 grade completed	160	34.638
9–12 grade completed	63	13.64
Diploma and above	22	4.76

### Prisoner’s prison environment related characteristics

From a total of 462 study participants, 53 (11.47%) are awaiting trial and 10 (2.16%) have been sentenced to life in prison. From the criminality type that the prisoner was sentenced for, 193 (41.8%) of total respondents were sentenced for murder. Among respondent prisoners who knew their sentenced year, 209 (51.9%) were sentenced for more than 5 years. One hundred ninety-seven (42.64%) of the prisoners refused to accept the reason for their incarceration. In addition, of those who knew their time in prison, 243 (59.41%) did not accept the total penalty year. The study participants’ median time in prison was 5.6 years (IQR = 2–15). Approximately 232 (50.22%) of prisoners were concerned about the difficulties they would face after being released from prison and 286 (61.9%) of the total respondents worked in prison. Participants with poor social support were 227 (49.13%).

### Clinical factors characteristics of prisoners

There were 133 (28.79%) study subjects who had chronic physical illness out of a total of 462 respondents and 42 (9.09%) of the respondents had a history of mental illness, in addition, 47 (10.17%) of study subjects had a family history of mental illness.

### Life time substance use characteristics of prisoners

According to the study, approximately half (50%) of the respondents had a history of lifetime alcohol use. About (41.99%) of the alcohol consumed was a local drink (“Tela”). Approximately 7.6% and 6.7% of respondents, respectively, had a lifetime history of chat and cigarette use.

### Prevalence of depression

**Fig. 2:**
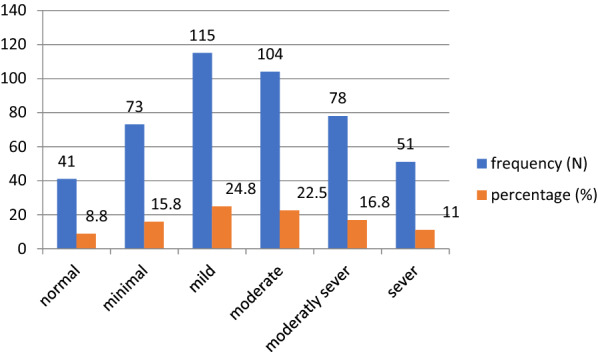
Levels of depression among prisoners in East Gojjam Zone prisons, 2019 (*N* = 462)

Based on the PHQ9 tool assessment, approximately half (50.43%) of study participants were identified as having depressive symptoms in the previous 2 weeks. In terms of depression, approximately (15.8%) of participants had minimal depression, while approximately (11.04%) of participants had severe depression (Fig. 2).

### Factors associated with depression among prisoners

Variables such as residency, work inside the prison, monthly income, history of mental illness, current chronic illness, total penalty year spent in prison, thinking about life after prison, and social supports had a *P* value less than 0.25 and were exported to multivariable analysis. In the final model, variables such as having work inside the prison, length of sentenced year, history of mental illness, income per month greater than 1500 birr, and not thinking about life after release had a *P* value less than 0.05 and were thus significantly associated with depression (Table 3).

**Table 3:** Factors associated with depression in the bivariable and multivariable analysis among prisoners in East Gojjam Zone prisons, Northwest Ethiopia, 2019 (*N* = 462)

Variables	Depression		COR (95% CI)	AOR (CI 95%)
	Yes	No		
Residency				
Rural	166	143	1.49 (1.01,2.2)	1.2 (0.74 ,1.8)
Urban	67	86	1	1
Work in prison				
Yes	133	153	0.66 (0.45, 96)	0.6 (0.37, 0 .96)**
No	100	76	1	1
History of mental illness				
Yes	31	11	1	1
No	202	218	0.328 (0.16, 67)	0.37 (0.16,0 .85)*
Chronic illness				
Yes	86	47	1	1
No	147	182	0.44 (0.29, 669)	0.64 (0.3, 1.3)
Sentenced year				
< 1	28	47	1	1
1–5	59	66	1.5 (0.84, 2.7)	1.72 (0.9, 3.2)
> 5	118	91	2.2 (1.3, 3.7)	2.2 (1.2, 4)*
Income in birr				
< 5000	170	145	1	1
500–1000	37	46	0.69 (0.42, 1.12)	0.69 (0.38, 1.2)
1001–1500	17	14	1.04 (0.49, 2.17)	0.57 (0.38, 1.2)
> 1500	9	24	0.32 (0.144,0 71)	0.16 (0.05, 0 0.5)*
Thinking about life				
Yes	144	88	1	1
No	89	141	0.38 (.265–0.56)	(0.27, 0.64)*

Bold emphasis indicates *P* < 0.05, **indicates *P* < 0.005

In this study, prisoners who worked inside the prison were 40% less likely to develop depression (AOR 0.6, CI (0.37–0.96) than those who did not work inside the prison. Inmates with no history of mental illness were 63 percent (AOR 0.37, CI 0.16–0.85) less likely to develop depression than those with a history of mental illness. Prisoners with a monthly income of more than 1500 birr were 84% (AOR 0.16, CI 0.05–0.5) less likely to develop depression than those with a monthly income of less than 500 birr. The odds of developing depression among prisoners who were sentenced for more than 5 years was 2.2 times higher (AOR 2.2, CI (1.2–4) as compared to those prisoners who were sentenced for less than 1 year. Moreover, the study indicated that prisoners who do not think about their life after released were 60% (AOR 0.4, (CI (0.27–0.64) less likely to develop depression as compared to those prisoners who thinks about their future life.

## Discussion

This institution-based cross-sectional study was carried out to determine the prevalence of depression and to identify risk factors for depression among prisoners in East Gojjam, Zone prisons. According to this study, the overall prevalence of depression among prisoners is 50.43% (CI 95%, 45,55%). This study’s findings are consistent with those of a previous study conducted in Bahir–Dar, Ethiopia (45.5%) [24].

However, this findings is higher than the studies conducted in Northwest Amhara (43.8%) [24], Jimma Town (41.9%) [8], in Nigerian (42.2%) [16], in Iran (29%) [15], and in France (17.9%) [14]. On the contrary the prevalence obtained from this study was lower than the studies done in Hawassa Town (56.4%) [17], Mekelle Ethiopia (55.9%) [26], and Malaysia (55.4%) [13]. The above variations could be explained by differences in the tool used to measure depression, the cut of value, the study settings, the length of sentenced year difference, and differences in the environmental characteristics of prisons.

In this study, prisoners who worked inside the prison were 56% less likely to develop depression than those who did not work inside the prison. The findings are consistent with previous research conducted in Brazil [31], Hawassa Central Prison [17], and Jimma Town Prisoner [8]. It is well known that working gives people pleasure in a variety of ways; it can be a way for them to spend their time, earn money, support their families, and live a better life both inside and outside of prison. Prisoners who had no past history of mental illness were 63% less likely to develop depression as compared to those who had history of mental illness. The finding is similar with study in Hawassa Central prison, Ethiopia [17], in Nigeria [16], and in France [14]. The possible reason is might be due to that prison environment can easily stimulates past mental health problems and leads to depression. When compared to convicts with a monthly income of less than 500 Ethiopian birr, individuals with a monthly income of more than 1500 Ethiopian birr were 84% less likely to develop depression. The likely answer is that having enough birr is utilized for many things, and inmates want to go about their daily lives as usual, and they need things such as food, clothing, and as much as possible to maintain their families and their lives in and outside jail which could reduce stress. Supportive findings are documented in Hawassa, Ethiopia [17] and Kenya [32].

The odds developing depression among prisoners who are sentenced for more than 5 years was 2.2 times higher as compared to prisoners who are sentenced for < 1 year. The possible explanation for this is that as the length of time spent in jail increases, convicts may become more concerned about their future. Furthermore, life in prison inhibits their freedom of movement, separation from their loved ones, denial of sexual intimacy, and many other aspects of their lives that are not typical. As a result, staying in prison for a longer period of time will make you depressed. These finding was supported by studies done in Bahir–Dar Town [24], and United Kingdom [33].

Furthermore, this study found that prisoners who did not consider life difficulties after prison were 60% less likely to develop depression than those who did consider life difficulties after prison. This can be explained by the fact that, physiologically, more thinking can lead to anxiety, stress, and depression. When prisoners consider their life after release from prison, they may be concerned about a variety of issues. Because they are incarcerated, their home, family, and resources may be lost, and starting a new life may be the main challenge, so prisoners may develop depression when they consider the difficulties of life after incarceration. Supportive findings are documented in Northwest Amhara [19], and in Jimma town prison, Ethiopia [8].

In resource poor countries, the magnitude of psychiatric disorders in prison populations is higher than in the general population. The prevalence could even be higher than in high-income countries. Because correctional facilities in low-income economies frequently lack basic treatment resources, the implementation of cost-effective interventions and scalable treatments for individuals suffering from depression is required.

**Conclusions**

According to this survey, half of the inmates in East Gojjam prisons suffer from depression. In comparison to their counterparts, convicts who had no work in jail, had a history of mental illness, had a lower monthly income, and worried about life after prison were more likely to develop depression. According to the findings of this study, all prisoners who are severely depressed should be treated and assisted in their rehabilitation. Prisoners must be involved in work and given more opportunities to earn money. Prison administration officers must teach poisoners how to live their lives after they are released from prison. The assigned professionals in prison who provide counseling should address effective counseling, screening, and evaluating the depression status of the prisoners. It would be preferable if special training and counseling were provided for prisoners with a history of mental illness as well as those who have been incarcerated for an extended period of time. Nongovernmental organizations (NGOs), and the government would benefit from a greater emphasis on prisoner health.

## Limitations of the study

Due to the nature of the cross-sectional study design, the study had its own limitations; it does not identify the temporal relationship between associated factors and depression. Because the study was conducted only in prisons, it does not generalize to the general population. Some of the reports were based on prior events, which can lead to recall bias. Alcohol drinking, Khat chewing, and other substances are delicate topics that might lead to social desirability bias.

## Supplementary Information

**Additional file 1. **Data collection tool.

## Data Availability

All the data are available from the corresponding author up on a reasonable request.

## Abbreviations

AOR: Adjusted odds ratio
COR: Crude odds ratio
CI: Confidence interval
PHQ: Patient health questionnaire
WHO: World Health Organization
NG: Non-Governmental Organization
