# Supplementary material for: Depression and its associated factors among prisoners in East Gojjam Zone prisons, Northwest Ethiopia: a multi-centered cross-sectional study
Source: Eur J Med Res. 2022 Jul 30;27:136. doi: 10.1186/s40001-022-00766-0 (PMC9338580; doi:10.1186/s40001-022-00766-0)
Supplement: Supplementary file 1 — Additional file 1. Data collection tool. [file 40001_2022_766_MOESM1_ESM.docx]

1. **DATACOLLECTION TOOL**
   1. **In English Language**

| **Part one socio- demographic characteristics** | | | |
| --- | --- | --- | --- |
| Number | Questions | | Response |
| 1 | Identification number | | … |
| 2 | Sex | | 1.male 2. female |
| 3 | Age in year | | ……. |
| 4 | Religion | | 1. Orthodox 2. Muslim 3. Protestant 4. Catholic 5. Other(specify) |
| 5 | Education status | | 1.Can read and write  2. primary education  3.Secondary education  4. Diploma  5. Degree and above |
| 6 | Residence | | 1. Urban  2. Rural |
| 7 | Ethnicity | | 1.Amahara  2. Gurage  3. Oromo  4. Afar  5. Tigre  6. Other (specify……………) |
| 8 | Marital status | | 1. Married 3. Single 2. Divorced 4. widowed |
| 9 | Do you Have children? | | 1. Yes 2. No |
|  | Part two Prisoners prison environmental characteristics | | |
| 10 | Do you have a work in prison? | 1.Yes  2.No | |
| 11 | If you say yes in the above question number 10 specify the type of work you have | 1.Daily labor  2.Metal work  3.Wood work  4.Tailor  5.Weaver  6.Handiwork  7. Other (specify) | |
| 12 | Average income you get per month in birr? | …. birr | |
| 13 | Do you have a job before imprisonment | 1.yes  2.no | |
| 14 | Do you practice religious activity? | 1.yes  2.No | |
| 15 | Did you have history of mental illness before prisoned that you follow regularly in health institution? | 1.yes  2.No | |
| 16 | Do you have history of mental illness in your family | 1.yes  2.No | |
| 17 | Have you a known chronic disease you following in health facility that confirmed by Health professional? | 1.yes  2.No | |
| 18 | If you say yes in the above question, what type of chronic physical illness do you have? You can answer more than one answer. | 1.heart disease  2.Diabetes mellitus  3.Hypertension  4.Epelepsy  5. Other (specify) | |
| 19 | Have you got health services when you need? | 1.Yes  2. No  3.No I didn’t need till now | |
| 20 | Type of criminal you charged, specify? | 1.Robbery  2.Rape  3.Corruption  4. Murderer  5. Theft  6. Related to government  7.Fighting  8. Other (specify) | |
| 21 | How long is the time duration you stay/spent in prison until today? | …………. | |
| 22 | Total convicted/sentenced year | ………. month/ ……year | |
| 23 | Did you accept the crime that reasoned for prisoned? | 1.yes  2.No | |
| 24 | Did you accept the total convicted/sentenced year | 1. Yes  2. No | |
| 25 | Type of prisoner | 1. Convicted  2. Waiting trial  3. Life time prisoners | |
| 26 | Did you ever incarcerated before this incarceration in the past? | 1.yes  2.No | |
| 27 | If yes for question number 24 how many times you become incarcerated in the past? | __________times | |
| 28 | Do you think life after released from prison is difficult | 1.yes  2.No | |
| 29 | Did you use alcohol in your life time? | 1.yes  2.No | |
| 30 | If yes for question number 27what type of alcohol most frequently use in your life time? | 1. Beer  2. wine  3. Teje  4. Arekie  5. Tela  6. Other (specify) | |
| 31 | Did you use chat in your life time before prisoned? | 1.yes  2.No | |
| 32 | Did you use cigarettes in your life time before prisoned? | 1.yes  2.No | |
| 33 | Did you use other than the above substances or alcohols in your life time before prisoned, if yes mention it… | …… | |

| ^Part three፡- patient health questionnaire9 depression measurement scale. Over the last 2 weeks, how often have you been bothered by any of the following problems? (Use “ X” to indicate answer).^ | | | | | |
| --- | --- | --- | --- | --- | --- |
|  | Not at all | Several days | | More than half the days | Nearly every day |
| 1.a little interest or pleasure in doing things | 0 | 1 | | 2 | 3 |
| 2. Feeling down, depressed, or hopeless |  |  | |  |  |
| 3. Trouble falling or staying asleep, or sleeping too much |  |  | |  |  |
| 4. Feeling tired or having little energy |  |  | |  |  |
| 5. Poor appetite or overeating |  |  | |  |  |
| 6. Feeling bad about yourself or that you are a failure or have let yourself or your family down |  |  | |  |  |
| 7. Trouble concentrating on things, such as reading the newspaper or watching television |  |  | |  |  |
| 8. Moving or speaking so slowly that other people could have noticed. Or the opposite being so fidgety or restless that you have been moving around a lot more than usual |  |  | |  |  |
| 9. Thoughts that you would be better off dead, or of hurting yourself |  |  | |  |  |
| Part four**:** the following questions ask about how you experience social Relationship. The required is about your immediate personal experience and about How the people visited you in the prison. | | | | | |
| Oslo social support questions | | | Response | | |
| O1. How many people are so close to you that you?  can count on them if you have serious personal  Problems? (Choose one option) | | | 1/ None | | |
|  |  |  | 2/ 1 or 2 | | |
|  |  |  | 3/ 3-5 | | |
|  |  |  | 4/ More than 5 | | |
| O2. How much concern do people show in what you are? Doing? (Choose one option) | | | 5. A lot of concern and  Interest | | |
|  |  |  | 4.Some concern and interest | | |
|  |  |  | 3. Uncertain | | |
|  |  |  | 2. Little concern and interest | | |
|  |  |  | 1. No concern and interest | | |
| O3. How easy is it to get practical help from family or Relatives if you should need it? (Choose one option) | | | 5. Very easy | | |
|  |  |  | 4. Easy | | |
|  |  |  | 3. Possible | | |
|  |  |  | 2. Difficult | | |
|  |  |  | 1. Very difficult | | |

- 1. **አማርኛ መጠይቅ**.

ይህ በምስራቅ ጎጃም የማረሚያ ተቋማት እስረኞች ላይ የሚከሰተውን የመደበር ወይምመደበት ሁኔታ እና ተዛማጅነ መንስኤወችን ለማዎቅ የተዘጋጀ መጠይቅ ነው::

| ክፍል አንድ ስለ ማህበራዊ እና ስነ-ሕዝብ ባህሪ | | |
| --- | --- | --- |
| ተ.ቁ | ጥያቄ | መለስ |
| 1 | መለያ ቁጥር | ………. |
| 2 | ፆታ………..? | 1.ወንድ 2. ሴት |
| 3 | ዕድሜዎ ስንት ዓመት ነው | ……..ዓመት |
| 4 | እርስዎ የየትኛው ሃይማኖ ተከታይ ነዎት | 1. ኦርቶዶክስ 3.ፕሮቴስታንት  2. ሙስሊም 4. ካቶሊክ  5. ሌ ላ |
| 5 | የትምህርት ደረጃዎ | 1.ማንበብና መጻፍ የማይችል  2.ማንበብና መጻፍ የሚችል  3.1-8 ክፍል  4.4.9-12 ክፍል  5.ዲፕሎማ እና ከዛ በላይ |
| 6 | የመኖሪያ አድራሻ | 1.ከተማ 2. ገጠር |
| 7 | ብሄር | 1. አማራ 4. ኦሮሞ 2. አገው 5. አፋር 3. ትግሬ 6. ሌላ |
| 8 | የጋብቻ ሁኔታ | 1. የጋባ/ባች 3. የተፋታ/ች 2. ያላገባ 4. የሞተባት |
| 9 | ልጂ አለዎት | 1 .አዎ  2 .የለኝም |
|  | ክፍል ሁለት፡ የአስረኛ እና እስርቤት ሁኔታን በተመለከተ | |
| 10 | በማረሚያ ቤት ውስጥ ስራ አለዎት? | 1. አዎከ 2. የለኝም |
| 11 | መልስዎ አዎ ከሆነ ለ ጥያቄ ቁጥር 10 የስራ አይነት ይግለጹ | 1. የቀን ስራ  2.የበረታብረት ስራ 5. ሽመና  3. የእንጨት ስራ 6.የእጅ ስራ  4. ልብስ ሰፊ 7.ሌላ (ይግለጹ) |
| 12 | በወር ምንያህል ገቢ ያገኛሉ በብር ይግለፁ? | ……….ብር |
| 13 | ከመተሰርዎ በፊት ስራ ነበርዎት | 1.አዎ 2.የለም |
| 14 | ሃይማኖታዊ ስረአትን ማስሄድ/መሳተፍ ይችላሉ? | 1.አዎ እችላለሁ 2. አይ አልችልም |
| 15 | ከመታሰርዎ በፊት በጤና ተቋማት /በህክምና ባለሙያ የተረጋገጠ የዕመሮ ህመም ነበረብዎት | 1. አወ 2. የለም |
| 16 | አሁን በህክምና ተቋም ውስጥ የሚከታተሉት በህክምና የታወቅ ስር የሰደደ ወይም የቆዬ ህመም አለብዎት | 1.አዎ 2.የለም |
| 17 | ለጥያቄ ቁጥር 16 መልስዎ አዎ ከሆነ ምንድነ ነወ የተባሉት፡ይግለጹ | ­­­­­­­1. የልብ ህመም 2.ስኮር  3.የደም ግፊት 4.የሚጥል በሽታ  5.ሌላ ጥቀሱ….. |
| 18 | ከቤተሰብዎ ውስጥ የአዕምሮ ህመም ታሞ የሚያውቅ ሰው አለ | 1.አዎ 2.የለም |
| 19 | ለህመምዎ ህክምና ሲፈልጉ በቀላሉ አገለገሎት ያገኛሉ | 1. አዎ በጊዜው  2 .አላገኝም  3.አስፈልጎኝ አያውቅምሠ |
| 20 | የታሰሩበትን ምክንያት ምን ነበር ይግለጹ | 1. ዘረፋ 2.አስገድዶ መድፈር  3. ሙስና 4.ነፍስማጥፋት  5.ሌብነት 6. ፖለቲካዊ  7. ገጭት 8. ሌላ (ዝርዝር ይግለጹ) |
| 22 | ወንጀሉን መስራትዎ አምነው ተቀብለውታል | 1.አወ 2.አልተቀበልኩም |
| 23 | ለምን የህል ጊዜ ነው የተፈረደበዎት ውሳኔ ጊዜ | ……..ወር/ …………. አመት |
| 24 | የተፈረደብዎት የቅጣት ፍርድ ኣግባብ ነው ብለው ያመናሉ | 1.አዎ 2.አየደለም |
| 25 | የእስር ዓይነትዎ | 1. ፍርደኛ  2. ቀጠሮ ጠባቂ.  3.ደሜ ይፍታሽ |
| 26 | ከአሁን በፊት ታስረው ያውቃሉ | 1.አዎ 2.የለም |
| 27 | መለስዎ አዎ ከሆነ ስንት ጊዜከአሁን በፊት ታስረዋል ይገለጹ | ……………ጊዜ |
| 28 | ከእስር ከተፈታሁ በኋላ ህይወት አስቸጋሪ የሆናል ብለው ያስባሉ? | 1.አዎ 2.አላስብም |
| 29 | ከመታሰርዎ በፊት ሁልጊዜ አልኮልይ ይጠቀሙ ነበር | 1.አዎ 2.የለም |
| 30 | ለጥያቄ ቁጥር 29 መልስዎ አዎ ከሆነ ብዙ ጊዜ የሚወስዱት አልኮል ከሚከተሉት ውስጥ የቱ ነው? | 1. ቢራ 2. ወይን  3. ጠጅ 4. አረቂ  5. ጠላ 6.ሌላ………. |
| 31 | ከመታሰርዎ በፊት ሁልጊዜ ጫት ይጠቀሙ ነበር | 1.አዎ 2.የለም |
| 32 | ከመታሰርዎ በፊት ሁልጊዜ ሲጋራ ያጨሱ ነበር | 1.አዎ 2.የለም |
| 33 | ከመታሰርዎ በፊት ሁልጊዜ ከሌይከተጠቀሱት ውጭ ሌላ ይጠቀሙ ከነበር የጥቀሱ | ……… |

| ክፍል ሶስት ፡ ባለፉት ሁለት ሳምንታት, ከሚከተሉት ችግሮች ውስጥ በተደጋጋሚ ጊዜ የተከሰቱ ምልክቶች እርስዎ ላይ የትኞቹ ናቸው ? | | | | |
| --- | --- | --- | --- | --- |
|  | የለም | ብዙ ቀናት | ከግማሽ ቀን በላይ | ቀኑን በሙሉ ማለት ይቻላል |
| 1.ስራህን/ሽን ለመስራት ዝቀተኛ ፍላጎት መኖር ወይም ራስን በሚየዝናኑ ነገሮች ላይ አለመደሰት፤ወይም የሚያዝናኑ ነገሮች ለይ አለማሳተፍ | 0 | 1 | 2 | 3 |
| 2.የፍላጎት መቀነስ መደበር እና የተስፋ መቁረጥ ስሜት መኖር፡፡ |  |  |  |  |
| 3.የመወራጨት እንቅስቃሴ ማሳየት ወይም እንቅልፍ ለመውሰድ መቸገር፡፡ |  |  |  |  |
| 4.የድካም ስሜት ወይም የኃይል ማነስ ስሜት መኖር፡፡ |  |  |  |  |
| 5.የምግብ ፍላጎት መቀነስ ወይም ከልክ በላይ መመገብ |  |  |  |  |
| 6. ስለራስህ/ሽ መጥፎ ስሜት ወይም በራስ አለመተማመን ወይም እራስህንና ቤተሰብህን ዝቅ አድርጎ ማዬት፡፡ |  |  |  |  |
| 7. በነገሮች ላይ ትኩረት የማድረግ ችግር ፤ ለምሳሌ ጋዜጣ ማንበብ ወይም ቴሌቪዥን መመልከት የመሳሰሉትን ነገሮች ላይ ትኩረት ማነስ፡፡ |  |  |  |  |
| 1. ቀስ በቀስ ወይም ቀስ ብለው ሌላ ሰው እነደሚያዳምጠዎት ፤ልክ አጠገብዎ ሰው እንደለ በማሰብ ንግግረ ማድረግ፤ ወይም ወዲያና ወዲይ መወራጨት ከወትሮው የተለዬ ሁኔታ ማሳዬት |  |  |  |  |
| 9.መሞት ይሻላል ብለው አስበው ወይም ራሰስዎን ለመገደል ሞክረው ያውቃሉ |  |  |  |  |
| ድምር……………. | 1. ድምር1-4 ዝቅተኛ ድብርት  2. ድምር 5-9 መልስተኛ ድበርት  3. ድምር10-14 መካከለኛ ድብርት  4. ድምር 15-19 መካከለኛ ክባደ ድብርት  5.ድንር 20-27 ክባድ ድበርት | | | |

| ክፍል 4-የኦስሎ -3 ማህበራዊ ድጋፍ መለኪያ መጠይቅ፡ | መልስ | | | | | |  |
| --- | --- | --- | --- | --- | --- | --- | --- |
| O1. በጣም ከባድ ችግር ቢያጋጥምህ /ሽ ስነት ሰው በቁጥር በቅርብ ልታገኝ ትችላለህ/ሽ | 4/ ከአምስት በለይ | 3/  3-5 | 2/  1 or 2 | | | 1/  ምንም | |
| O2. ሰዎች አንተ/ች ስለምትሰራው/ትሰሪው ነገር ምንያህል ያስባሉ ወይም ይጨነቃሉ | 5/  ብዙ ያማሰብሉ | 4/  .በመጠኑ ያስባሉ | 3/ ዕርግጠኛ አይደለሁም | 2/  በትንሹ ማሰብ እና ፍለጎት | 1/  ምንም አያስቡም ወይም አይፈልጉም | |  |
| O3. እርዳታ ማግኘት ከጎረቤት ብያስፈልግህ/ሽ ምን ያህል ቀላል ነው | 5/  በጣም ቀላል | 4/  ቀላል | 3/  የቻላል | 2/ ይከብዳል | 1/  በጣም ይከብዳል | |  |
| ድመር…………………………………. | 1.3-8 አነስተኛ ማህበራዊ ድጋፍ  2.9-11 መካከለኛ ማህበራዊ ድጋፍ  3. 12-14 ጠንከራ ማህበራዊ ድጋፍ | | | | | |  |
